# Phylogenetic Diversity of T4-Type Phages in Sediments from the Subtropical Pearl River Estuary

**DOI:** 10.3389/fmicb.2017.00897

**Published:** 2017-05-18

**Authors:** Maoqiu He, Lanlan Cai, Chuanlun Zhang, Nianzhi Jiao, Rui Zhang

**Affiliations:** ^1^State Key Laboratory of Marine Environmental Science, Institute of Marine Microbes and Ecospheres, Xiamen UniversityXiamen, China; ^2^Department of Ocean Science and Engineering, South University of Science and TechnologyShenzhen, China

**Keywords:** Pearl River Estuary, sediment, *g23*, viral abundance, viral morphology

## Abstract

Viruses are an abundant and active component of marine sediments and play a significant role in microbial ecology and biogeochemical cycling at local and global scales. To obtain a better understanding of the ecological characteristics of the viriobenthos, the abundance and morphology of viruses and the diversity and community structure of T4-type phages were systematically investigated in the surface sediments of the subtropical Pearl River Estuary (PRE). Viral abundances ranged from 4.49 × 10^8^ to 11.7 × 10^8^ viruses/g and prokaryotic abundances ranged from 2.63 × 10^8^ to 9.55 × 10^8^ cells/g, and both decreased from freshwater to saltwater. Diverse viral morphotypes, including tailed, spherical, filamentous, and rod-shaped viruses, were observed using transmission electron microscopy. Analysis of the major capsid gene (*g23*) indicated that the sediment T4-type phages were highly diverse and, similar to the trend in viral abundances, their diversity decreased as the salinity increased. Phylogenetic analysis suggested that most of the *g23* operational taxonomic units were affiliated with marine, paddy soil, and lake groups. The T4-type phage communities in freshwater and saltwater sediments showed obvious differences, which were related to changes in the Pearl River discharge. The results of this study demonstrated both allochthonous and autochthonous sources of the viral community in the PRE sediments and the movement of certain T4-type viral groups between the freshwater and saline water biomes.

## Introduction

Marine sediments cover around 70% of the earth’s surface and make a considerable contribution to ecological processes and biogeochemical cycling in the ocean. They are colonized by a vast range of largely unknown microorganisms (Tyler, 2003; Danovaro et al., 2014). Generally, microbes are more abundant in sediments than in seawater. Viruses, the most abundant life forms on this planet, have been confirmed to play an important role in controlling the microbial community structure, promoting biological evolution and nutrient circulation, and maintaining the energy flow (Fuhrman, 1999). In addition, viruses are one of the main repositories of genetic diversity in the world (Weinbauer and Rassoulzadegan, 2003).

Viruses tend to be more plentiful than their hosts in sediments, with abundances ranging from 10^8^ to 10^9^ viruses/g (Danovaro et al., 2008a). Previous studies carried out in deep sea sediments worldwide have shown that most prokaryotic deaths in sediments were due to viral lysis, resulting in the release of between 0.37 and 0.63 Gt C/year globally, and that the resulting organic detritus was an important trophic resource for the metabolism of non-infected microbes (Danovaro et al., 2008b; Dell’Anno et al., 2015). These findings indicate that sediment viruses can influence biogeochemical cycles in fundamental ways (Danovaro et al., 2014; Corinaldesi, 2015). One of the relatively common observed marine viruses are the tailed phages, which can be divided into myophages, podophages, and siphophages according to their tail structure, and T4-type phages are a major group of myophages (Wommack and Colwell, 2000; Suttle, 2005, 2007; Hurwitz and Sullivan, 2013). The *g23* gene, the major capsid gene of the T4 phage, which infects *Escherichia coli*, is widespread in the genomes of the T4-type phages because of its conserved sequences (Tetart et al., 2001; Desplats and Krisch, 2003). Filée et al. (2005) found that the phylogenetic analysis of the T4-type phages based on the *g23* gene was consistent with that based on several conserved genes. In addition, they obtained certain novel *g23* sequences from environmental samples, and classified them into five new marine groups (Marine Groups I–V) (Filée et al., 2005). Subsequently, *g23* sequences retrieved from paddy soils were grouped into nine paddy groups (Paddy Groups I–IX) (Fujii et al., 2008; Wang et al., 2009b). T4-type phages were found to be highly diverse in different environments, including freshwater lakes (López-Bueno et al., 2009; Butina et al., 2010), black upland soil (Liu et al., 2011; Wang et al., 2011), Arctic glacier (Bellas and Anesio, 2013), and wetlands (Zheng et al., 2013). These studies also suggested that the *g23* gene was a good indicator of the diversity of T4-type phages in many kinds of environments (Adriaenssens and Cowan, 2014). However, the *g23* gene has not been used in investigations of viruses in sediment environments.

As one of the most productive environments on Earth (Bunt, 1975), estuaries have both salinity and trophic gradients because of the mixing of nutrient rich freshwater inputs from rivers and relatively nutrient depleted seawater (Hewson et al., 2001). Microbial processes in both the water column and sediments in estuaries mediate the transfer of nutrients, including nitrogen, phosphorus, and carbon, from land to sea. As a result of these processes, a huge amount of carbon dioxide is released into the atmosphere and while particulate or dissolved organic carbon is stored in water column or sediments (Bauer et al., 2013). However, studies of benthic microbes, especially viruses, in sediments are limited.

The Pearl River Estuary (PRE), the second largest estuary in China, is an important linking ecosystem between the mainland and the South China Sea (Cai et al., 2004). Previous studies confirmed that river inputs contributed to the development of microbial communities in the PRE (Zhou et al., 2004; Owen, 2005) and that microbial diversity differed between freshwater and saltwater regions (Jiang et al., 2009; Qiu et al., 2010; Xie et al., 2014). Liu et al. (2014) reported that *Deltaproteobacteria, Thermoplasmata*, and Marine Group I were more abundant in saltwater sediments, whereas *Chloroflexi, Spirochaetes, Betaproteobacteria*, and methanogens were more prevalent in freshwater sediments. Spatial variations of benthic ammonia oxidizing archaea and bacteria were also revealed and related to the environmental gradient of the PRE (Chen et al., 2012). However, there is very little information about the benthic viral community of the PRE. In the present study, therefore, to fill this knowledge gap and to gain an improved understanding of benthic microbial ecology of this subtropical estuary, the abundance, morphology, and diversity of the viriobenthos were investigated. As far as is known, this is the first study that has investigated the diversity of the T4-type phages in marine sediments.

## Materials and Methods

### Sample Collection

**FIGURE 1:**
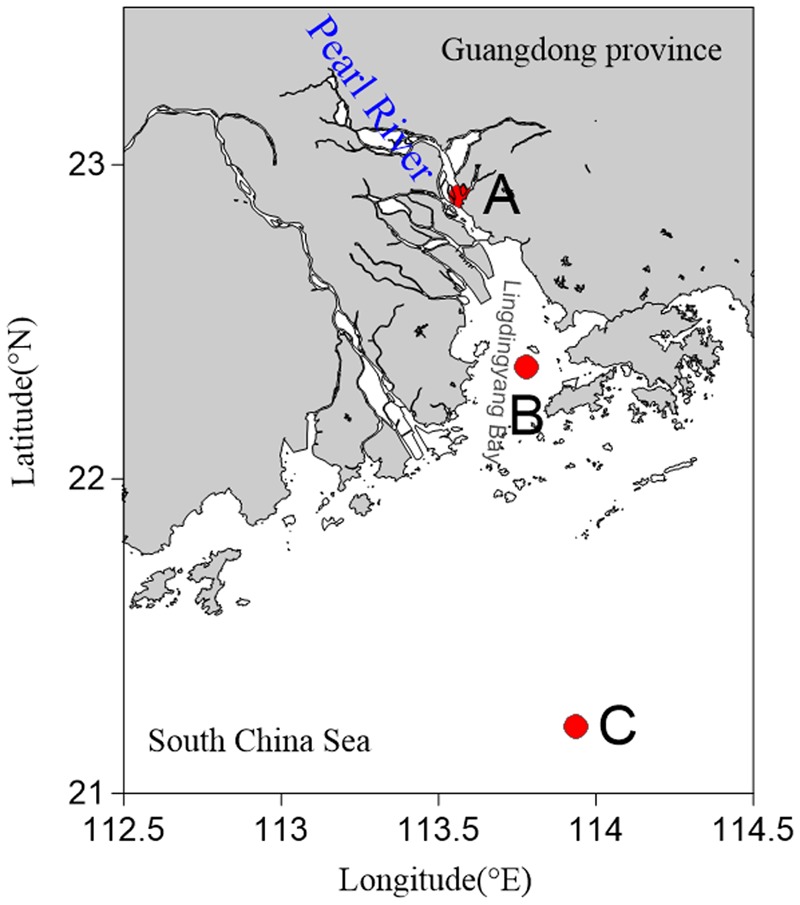
**Map showing sampling sites in the Pearl River Estuary. (A)** Freshwater site; **(B)** brackish water site; **(C)** saltwater site.

Sediments were sampled at three stations in the PRE during July and August 2013. These stations were distributed from the river mouth to the open sea along a gradient of increasing depth and salinity (**Figure 1**). Samples were collected on the vessel using a sediment core sampler (KC Kajak, Denmark). The surface layer of the sediment samples was scraped off and the remaining sediments were placed into sterile ziplock bags. The samples were then stored at -80°C until further analyses were carried out. Temperature, salinity, and dissolved oxygen (DO) were measured *in situ* using a YSI Professional Plus multiparameter meter (YSI Incorporated, Yellow Springs, OH, United States).

### Enumeration of Microorganisms

**FIGURE 2:**
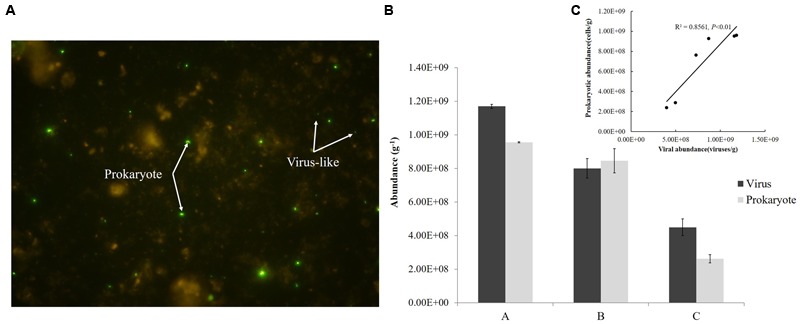
**Fluorescence imaging (A)**, abundances **(B)**, and correlation **(C)** of viruses and prokaryotes in sediments from the Pearl River Estuary.

Viral and prokaryotic abundances were determined by epifluorescence microscope according to Danovaro and Middelboe (2010) with a few modifications. Briefly, sediment (1.0 g) was transferred into sterile 50 mL centrifuge tubes. After adding 9 mL of SM buffer (50 mM Tris-HCl, 100 mM sodium chloride, 10 mM magnesium sulfate heptahydrate, 0.01% gelatin, pH = 7.5) and glutaraldehyde (0.5% *v/v*), the samples were shaken violently for 15 min with a vortex shaker (Mobio Vortex-Genie 2, MoBio) in the dark. Sodium pyrophosphate solution (5 mM final concentration) was then added and the slurry was shaken for 15 min. Sonicating treatment was also applied to optimize the extraction (Danovaro and Middelboe, 2010). Subsamples were diluted 100–500 times. Aliquots of the subsamples were stained with SYBR Green-I and filtered on Anodisc aluminum oxide filters (0.02 μm pore size). The filters were analyzed using epifluorescence microscopy (Olympus BX51) and the microbial particles were observed under blue light (wavelength of 365 nm) (**Figure 2A**). Between 10 and 50 fields were viewed for viruses, and a minimum of 300 cells were counted for prokaryotes. Each sample was measured three times and fluorescent signals in the fields were counted under the same conditions.

### Morphology of Virus-Like Particles

Subsamples produced by the counting process were centrifuged at a low speed and the supernatants containing virus-like particles were collected. The supernatants were then precipitated using polyethylene glycol (PEG-8000; 10% *w/v*) and incubated overnight at 4°C. Virus-like particles from the PEG pellets were suspended in 2 mL of SM buffer. The resuspended particles were layered on the top of caesium chloride step gradients (1.7, 1.5, and 1.35 g/mL layers in SM buffer), followed by spinning in a Optima L-100 XP Ultracentrifuge (Beckman Coulter) at 34,100 rpm for 3 h at 4°C, using a SW 41 Ti swinging bucket rotor. Virus-like particles were collected from the 1.35–1.5 g/mL fraction and confirmed by epifluorescence microscopy. A portion (10 μL) of a mixture of different viral extracts was attached to Formvar carbon coated copper electron microscopy grids (200 mesh) for 20 min. Virus-like particles were negatively stained with 1% (*w/v*) phosphotungstic acid for 1 min. Excess stain was removed and the grids were dried on a filter paper. Grids were examined in a JEM-2100 electron microscope (Jeol, Akishima, Japan) operated at 80 kV at a magnification of 15,000–150,000×. Images were collected using a charge-coupled device image transmission system (Gatan, Inc).

### DNA Extraction, PCR, Cloning, and Sequencing

Total deoxyribonucleic acid (DNA) was extracted from 0.5 g of sediment samples using the FastDNA SPIN Kit for Soil (Qbiogene, Inc., Carlsbad, CA, United States) following the manufacturer’s protocol (Wang et al., 2009a). The *g23* genes of the T4-type phages were amplified using the degenerate primers MZIA1 *bis*(5′-GAT ATT TGI GGI GTT CAG CCI ATG A-3′) and MZIA6 (5′-CGC GGT TGA TTT CCA GCA TGA TTT C-3′) (Filée et al., 2005). A portion (1 μL) of each template was added to 25 μL of polymerase chain reaction (PCR) mixture containing ExTaq buffer (without Mg^2+^; Takara Bio, Shiga, Japan), 2.5 mM magnesium chloride, 0.2 mM of each deoxyribonucleoside triphosphate polymerase, 10 pmol of each of the primers, 0.08% bovine serum albumin (Takara), and 2.5 U ExTaq DNA polymerase (Takara) (Fujihara et al., 2010). Negative controls contained all the reagents and sterile water instead of a template. PCRs were performed with the following PCR cycle parameters: denaturation at 94°C for 5 min, 35 cycles of denaturation at 94°C for 45 s, annealing at 50°C for 60 s, extension at 72°C for 45 s, and a final extension at 72°C for 10 min (Filée et al., 2005). Amplification products were run in a 1% agarose gel. Bands of the expected size were excised and purified using an agarose gel DNA purification kit (Takara). The PCR products were cloned into the pMD18-T vector (Takara) and then transformed into competent cells of *Escherichia coli* DH5α. Positive clones were screened using PCR re-amplification with vector primers M13-F/M13-R and randomly selected for sequencing using an automated DNA sequence analyzer with BigDye Terminator chemistry (ABI model 3730, Applied Bio Systems, Perkin-Elmer).

### Phylogenetic Diversity Analysis

Sequences of the *g23* gene were grouped into operational taxonomic units (OTUs) based on a 3% sequence divergence cut-off using the DOTUR program (Schloss and Handelsman, 2005). Rarefaction and phylotype richness estimators (Chao1 Shannon and Simpson indices) for each clone library were also calculated using DOTUR. DNA sequences were translated to amino acid sequences using the EMBOSS Transeq program on the European Bioinformatics Institute (EBI) website. All sequences were checked for their closest relatives using a BLAST search at the amino acid level on the National Center for Biotechnology Information (NCBI) website. Neighbor-joining trees were constructed using Molecular Evolutionary Genetics Analysis software (MEGA 6.0) with 500-fold bootstrap support. DNA sequences of the *g23* clones were deposited in GenBank under accession numbers KX789788 to KX790242.

### Statistical Analysis

The histograms and correlation analyses were completed with SPSS software.

## Results and Discussion

### Environmental Characteristics

Sediment samples and overlying water were analyzed for environmental parameters including temperature, salinity, and DO (Supplementary Table 1). The salinity ranged from 0.14 to 36.06 in surface water, and showed a tendency to increase from Site A to Site C. Although the salinity (31.50) at Site C was lower in sediments than in the upper water (36.06), it was considered that Sites A, B, and C were regions of freshwater, brackish water, and saltwater, respectively. Among three sites, water depth at Site C is more than 70 m and the depths of Site A and Site B are similar (∼10 m). The tides of the PRE belonged to the mixed tide, mainly semidiurnal, and the tidal amplitude varied from 2.2 to 3.1 m during spring tide and from 0.6 to 1.1 m during neap tide (Wang and Zhu, 2012). Furthermore, previous studies have confirmed that coarse grained sediments (grain size > 2 μm) were concentrated at the upstream estuary and silty clay sediments were present in the middle and outer part of the PRE (Carman et al., 2007; Li et al., 2011), which was in agreement with the sediment types that we found.

### Abundance of Viruses and Prokaryotes

Abundance of viruses was 1.17 × 10^9^, 8.0 × 10^8^, 4.49 × 10^8^ viruses/g from Sites A, B, and C, respectively and that of prokaryotes was 9.55 × 10^8^, 8.46 × 10^8^, 2.63 × 10^8^ cells/g, respectively; both decreased from freshwater to saltwater (**Figure 2B**). This observation was comparable to viral abundances obtained from an estuarine area in the Coral Sea (Australia; 6.7–14.4 × 10^8^ viruses/g of sediment) (Hewson et al., 2001) and Chesapeake Bay (United States; 3.4–8.1 × 10^8^ viruses/g) (Drake et al., 1998). Simultaneously, prokaryotic abundances in sediments from the PRE were more than two orders of magnitude greater than the abundances in the overlying water (10^6^ cells/mL) (Ni et al., 2015) and were similar to those found in sediments of the Coral Sea and along the coast of Chile, where abundances of prokaryotes up to 10^8^ cells/g were reported (Hewson et al., 2001; Middelboe and Glud, 2006). In this study, the abundances of virus-like particles and prokaryotes were significantly correlated (*P* < 0.01) (**Figure 2C**) and the viruses to prokaryotes ratio (VPR) at the three sites ranged from 0.95 to 1.71. Generally, the relationship between the viral and prokaryotic abundances in the sediments is not well-defined (Danovaro et al., 2008a). Values of VPR reported for marine sediments ranged from 0.001 in deep marine sediment, 200 m below the sea floor, to 225 for a marine subsurface sediment, with a mean of 12.1, which were higher than values obtained in this research (Parikka et al., 2016). However, viral counts were lower than prokaryotic counts in coastal sediments from Ancona Port (Italy), the Gulf of Thermaikos, and the Adriatic Sea, where VPR ranged from 0.2 to 0.9 (Mei and Danovaro, 2004). Low VPR were the result of either relatively low viral abundances or high prokaryotic abundances, or both. In the complex environment of the PRE, the VPR or the standing stock of viruses and prokaryotes in sediment depends on their source and fate, which are related to the physical, chemical, and biological settings of the PRE. This topic however deserves further investigation.

There are two possible origins of viruses in PRE sediments, allochthonous and autochthonous production (Hewson and Fuhrman, 2003). Suspended particles introduced by rivers adsorb a large number of viruses, then sink because of gravity, and then become an important source of the estuarine viriobenthos. The Pearl River supplies about 85 × 10^6^ tons of sediments annually, 85% of which are deposited in the estuary (Zhao, 1990; Zhou et al., 2004), meaning that the sedimentation rate of the PRE is between 10 and 18 mm/y (Owen, 2005). The concentration of suspended particulate matter tends to decrease from freshwater to saltwater because of dilution, contributing to a decrease in viral abundance from Site A to Site C. Meanwhile, the high abundance and activity of the hosts would lead to high viral production, which is significantly correlated with nutrient levels in the environment. Previous surveys of the spatial distribution of benthic viruses indicated a possible causal relationship between the benthic viral abundance and trophic state (Hewson et al., 2001; Danovaro et al., 2002, 2008a). In the PRE, the total organic carbon and nutrient concentrations, including phosphate (PO_4_^3-^) and dissolved inorganic nitrogen, were reported to decrease from freshwater to saltwater sediments (Liu et al., 2014). These reductions resulted in decreased abundance, possible decreased activity of prokaryotes, and subsequently, reduced autochthonous viral production.

### Morphology of Virus-Like Particles

**FIGURE 3:**
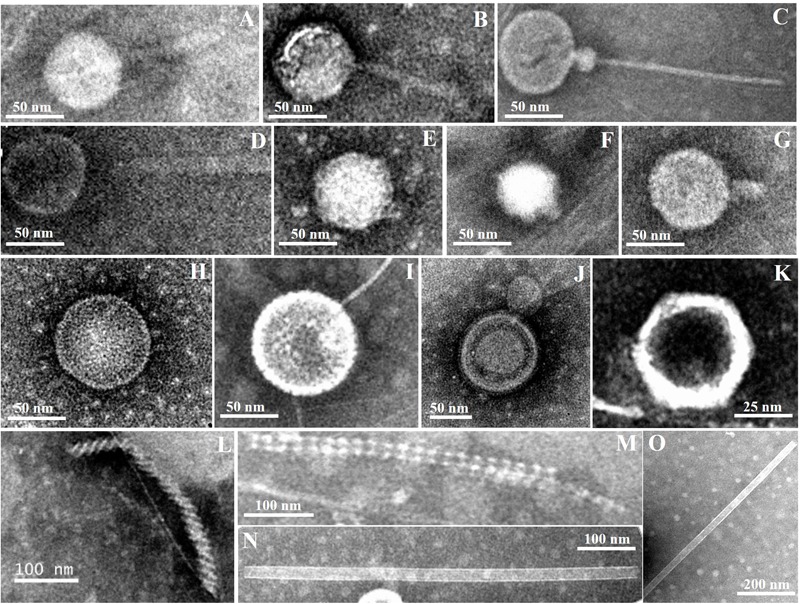
**Transmission electron micrographs of viral particles in Pearl River Estuary sediments.** Possible viral taxonomy was based on morphology: **(A,B)** (*Myoviridae*); **(C,D)** (*Siphoviridae*); (**E**–**G)** (*Podoviridae*); **(H,I)** (*Globuloviridae*); **(J)** (*Plasmaviridae*); **(K)** (*Rhizidiovirus*); **(L,M)** (*Inoviridae Plectrovirus*); **(N,O)** (*Rudioviridae*).

Diverse viral morphotypes were observed in the Pearl River estuarine sediments. In addition to common tailed viruses (**Figures 3A–G**), a number of spherical (**Figures 3E,H,I**), filamentous (**Figures 3L,M**), and rod-shaped viruses (**Figures 3N,O**) were also observed. Spherical viruses may belong to the *Globuloviridae* family, which comprise an envelope with helical symmetry and a superhelical nucleoprotein core containing linear double-stranded DNA (Haring et al., 2004). The majority of filamentous bacteriophages mostly infecting gram-negative bacteria belong to the *Inoviridae* family and they have been reported to be single-stranded DNA viruses (Day, 2011). Rod-shaped viruses should be classified into *Rudiviridae* family (Prangishvili et al., 1999).

To date, a few studies have reported that viral morphology was more diverse in sediment than in the water column (Danovaro and Serresi, 2000; Middelboe et al., 2003; Bettarel et al., 2006). Middelboe et al. (2003) found filamentous forms of viruses with both helical and icosahedral symmetries in sediments of the Nivå Bay (Zealand, Denmark), and reported that the number of the former was greater than that of the latter. However, filamentous and rod-shaped viruses are seldom observed in the water column (Brum et al., 2013). Therefore, the presence of these viral morphotypes may be an indication of autochthonous production by hosts specific to the sedimentary environments. Previous studies have also shown that siphoviruses and myoviruses accounted for the majority of viruses in sediments of the Baltic Sea (Jakubowska-Deredas et al., 2012). Different sample treatments may have some influences on observations. For example, violent sample treatments, including sonication, vibration, and centrifugation, may break the tails of most tailed viruses, leading to an observation that more non-tailed viral morphotypes were present in the samples. Quantitative transmission electron microscopy (qTEM) was applied to quantify viral morphology in sea water, and it was found that four viral morphotypes containing myoviruses, podoviruses, siphoviruses, and non-tailed viruses occupied the major part (Brum et al., 2013). Although there have been no similar studies of sediment viruses, myoviruses are one of the commonly observed viral groups in sediments from various marine and freshwater environments, including the PRE. Therefore, the diversity of myoviruses were investigated using the *g23* gene to try to explore the change of sedimentary T4-type viral communities along the estuarine gradient.

### Diversity of T4-Type Phages

In total, 455 viral *g23* clones were obtained from clone libraries and the number of OTUs was 96, 81 and 58 for Sites A, B, and C, respectively, identified with a cut-off of 97% nucleotide identity (**Table 1**). All the diversity indices, including the Shannon index (H′: 3.7–4.3), the Simpson index (1/D: 35.1–75.0), and Chao1 index (75.3–252.6), decreased from Site A to Site C. The decreasing trend of viral *g23* diversity from freshwater sediments to saltwater sediments in the PRE was similar to the trend in the viral abundances and, to some extent, suggests a positive relationship between viral population size and diversity. The amino acid identity of the closest relatives of the *g23* clones of the three sites ranged from 50 to 100%, with the majority having an identity of between 50 and 79% (Site A: 53.1%; Site B: 69.2%; Site C: 75.9%) (Supplementary Table 2). Furthermore, the majority of the clones were close to sequences from uncultured viruses and only four clones (two from Site B and two from Site C) were similar to the *g23* genes from cultured cyanophages. This indicates that there were numerous novel T4-type phages in the PRE sediments. In addition, the rarefaction curves of the three clone libraries (Supplementary Figure 1) indicated that the sampling size was still insufficient to capture the true diversity, and one may expect higher T4-type viral diversity in PRE sediment than observed in the present study. High throughput next generation sequencing for both PCR-amplified marker genes (e.g., *g23*) and metagenomic samples is needed to reveal sedimentary viral diversity in the future.

**Table 1:** Diversity indices of viral *g23* clone libraries recovered from the sediment sampling sites in the Pearl River Estuary.

Site	N	No. of OTUs^a^	H′	1/D	Chao1
A	150	96	4.3	75.0	252.6
B	150	81	4.1	59.2	201.5
C	155	58	3.7	35.1	75.3

*N, number of clones sequenced.*

*^a^OTU was defined as a 3% divergence at the DNA level.*

The 50 most abundant OTUs included 263 *g23* clones (57.8%), of which 25 OTUs were site-specific, whereas two OTUs were present at all three sites (Supplementary Figure 2). Eight OTUs (28% of the Site A clone library), which were mostly similar to those from paddy soil (six OTUs) (Fujii et al., 2008; Liu et al., 2011) and Donghu Lake (2 OTUs) (Huang et al., 2011), only appeared at Site A. Six OTUs appeared exclusively at Site B, accounting for 20.7% of the Site B clone library, and all of them were similar to sequences with 64–91% identity obtained from the Pacific Ocean (five OTUs) (Filée et al., 2005) and the Sargasso Sea (one OTU) (Goldsmith et al., 2015). Eleven OTUs, accounting for 31.3% of the Site C clone library, were specific to Site C, and six of them were also close to marine environmental *g23* sequences (Filée et al., 2005; Jamindar et al., 2012). Detailed information is provided in Supplementary Table 3. There were more OTUs in common between Site B and Site C (21 OTUs) than between Site A and the other sites (six OTUs), indicating that there was greater similarity in the viral communities in the sediments of Site B and Site C that are more influenced by seawater. Only OTUs 2 and 46 were common to all three sites. OTU 2 was 74% identical to the *g23* sequence from the Pacific Ocean (AAZ17601) that was shown to be in the Exo-T-even group. As reported previously, viruses belonging to the Exo-T-even group are cyanophages and existed widely, so it was not unexpected to find sequences close to this group (Filée et al., 2005; Fujii et al., 2008; Cahyani et al., 2009). OTU 46 was 73% identical to the clone sequence from the paddy soil in Japan (BAF52897) (Fujii et al., 2008), which might have been introduced to the sediments from river discharge. The wide distribution of these two OTUs with distinct origins in contrasting sediment environments of the PRE demonstrates that certain groups of T4-like myoviruses were transferred between biomes and that the estuary is a suitable site for the investigations of virus movements. Abundant viral populations that originated from different biomes in the same environment increase the opportunities for genetic exchange among viruses and their hosts (Bellas and Anesio, 2013) and, therefore, estuarine sediment environments may serve as one of the hotspots for viral exchange.

**FIGURE 4:**
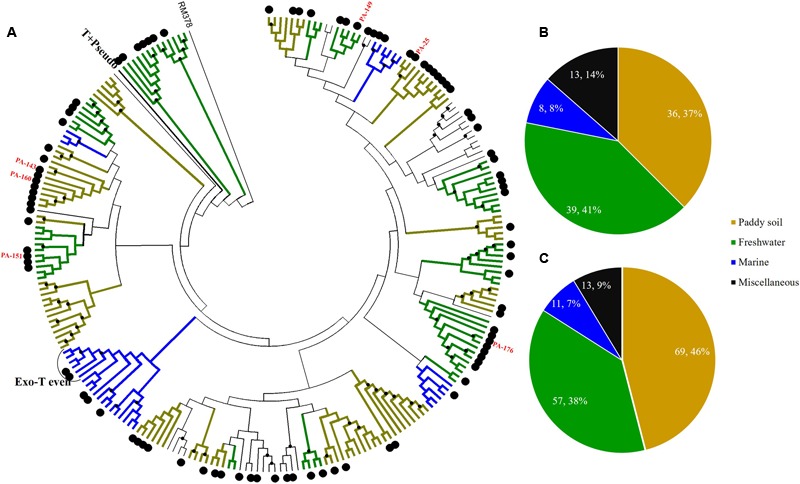
**Phylogenetic analysis of *g23* OTUs amino acid sequences obtained at Site A with those obtained from marine water, freshwater lakes, and paddy soils (A)**, and number of *g23* OTUs **(B)**, and clones **(C)** from Site A in different environmental groups. In the phylogenetic analysis, the large black dots indicate sedimentary sequences and the small black dots indicate internal nodes with a >50% bootstrap support. Different colored branches indicate *g23* groups from different environments: marine water (blue), freshwater lakes (green), and paddy soils (yellow).

**FIGURE 5:**
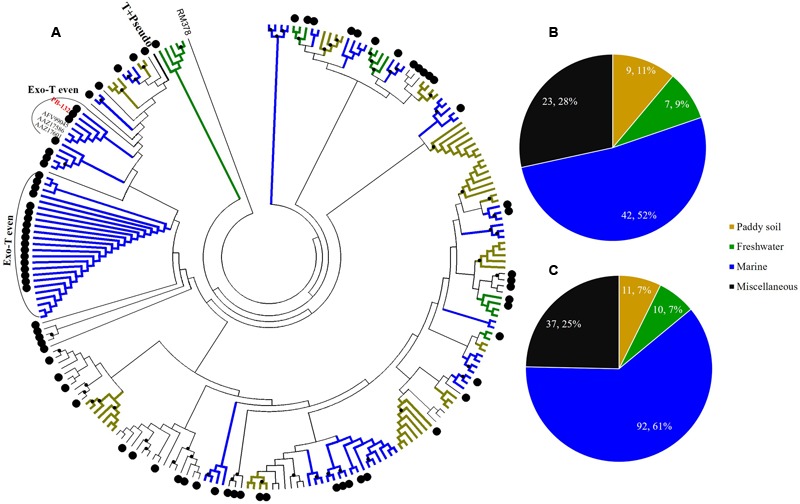
**Phylogenetic analysis of *g23* OTUs amino acid sequences obtained at Site B with those obtained from marine water, freshwater lakes, and paddy soils (A)**, and number of *g23* OTUs **(B)**, and clones **(C)** from Site B in different environmental groups.

**FIGURE 6:**
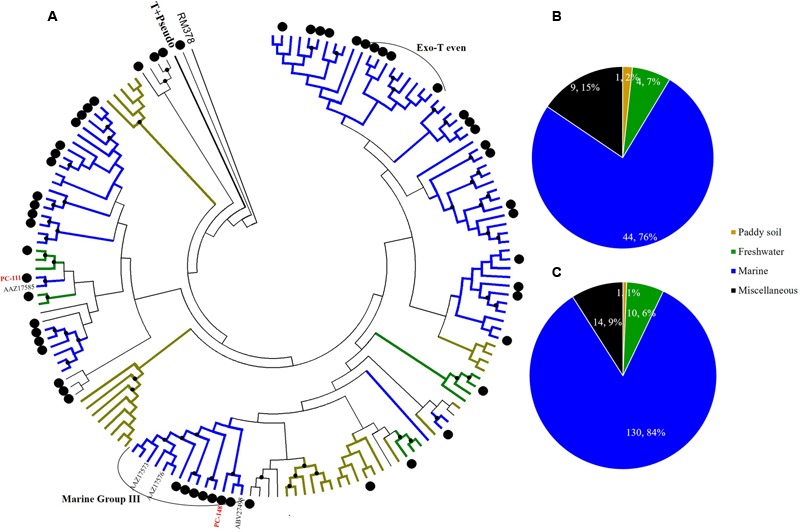
**Phylogenetic analysis of *g23* OTUs amino acid sequences obtained at Site C with those obtained from marine water, freshwater lakes, and paddy soils (A)**, and number of *g23* OTUs **(B)**, and clones **(C)** from Site C in different environmental groups.

Sediment *g23* OTUs from Site A, Site B, and Site C were compared with *g23* amino acid sequences obtained from marine water (Filée et al., 2005), freshwater lakes (López-Bueno et al., 2009; Butina et al., 2010), and paddy soils (Jia et al., 2007; Fujii et al., 2008; Wang et al., 2009a) respectively (**Figures 4–6**). These phylogenetic trees also included *g23* genes from phage RM378 and other phages classified into T-even and Pseudo T-even groups (“T+Pseudo” group in trees) (Tetart et al., 2001; Desplats and Krisch, 2003). Most of the *g23* OTUs from the PRE sediments closely resembled sequences from terrigenous (paddy and lake) and marine groups, which, to a certain extent, suggested that the overlying water was the main source of sedimentary T4-type phages (**Figures 4–6**). As mentioned previously, sedimentation of viruses that are adsorbed to suspended particles in the water column and autochthonous viral reproduction on the eroded matter from sediments are possible major sources of estuarine sediment viruses. Furthermore, as the sampling sites transitioned from the freshwater to the saltwater region, the majority of *g23* OTUs and clones in sediment samples also shifted from those associated with terrigenous groups (paddy soil and freshwater lake) to those associated with marine groups.

At freshwater Site A, most OTUs (78.1%) were affiliated with the terrigenous group (freshwater lakes 39/96, 41%; paddy soils 36/96, 37%), and those belonging to the miscellaneous group consisted of sequences from several environments (13/96, 13.5%) and the marine group (8/96, 8.3%) (**Figure 4B**). In addition, the relative abundance of terrigenous *g23* clones from Site A was 84%, which was much higher than those in the marine and miscellaneous groups (**Figure 4C**). The three most abundant OTUs of Site A (OTU PA-25, 12 clones; PA-143, 8 clones, and PA-160, 6 clones, Supplementary Table 4) were clustered into paddy soil branches, and had amino acid similarity of between 67 and 78% with *g23* sequences from paddy soil of Japan and north-east China (Fujii et al., 2008; Liu et al., 2012). Other abundant OTUs, namely PA-149 (six clones), PA-151 (four clones), and PA-176 (three clones), were associated with freshwater lake branches and shared between 75 and 88% amino acid similarity with the sequences from Annecy Lake (France) and Kotokel Lake (Russia) (Butina et al., 2013; Zhong and Jacquet, 2014). Similar *g23* sequences detected from geographically widespread, distinct and disconnected soil and lake environments suggested that these T4-type phages may be distributed globally.

At Site B, the brackish water site, more than half of the OTUs (42/81, 51.9%) were associated with the marine group, with fewer found in the miscellaneous (23/81, 28.4%) and terrigenous groups (16/81, 19.8%) (**Figure 5B**). The relative abundances of the marine group (61.3%) were also higher than the abundances of the miscellaneous (24.7%) and terrigenous groups (14%) (**Figure 5C**). Furthermore, all the abundant OTUs that represented more than five clones were clustered into the marine group. Among them, the most abundant, OTU PB-132 with 12 clones (Supplementary Table 4), was clustered with *g23* sequences from Delaware Bay (AFV99045) and the Pacific Ocean (AAZ17601, AAZ17586) that were previously classified into the Exo-T-even group (Filée et al., 2005) and shared 74% amino acid similarity. At Site C, the saltwater site, more than 75% of the OTUs belonged to the marine group (44/58), whereas only nine and five OTUs fell into the miscellaneous (15.5%) and terrigenous (8.6%) groups, respectively (**Figure 6B**). Marine group *g23* clones accounted for 84% of the relative abundance in the Site C clone library, which was also the highest out of the three sites (**Figure 6C**). In the phylogenetic tree of Site C, OTU PC-111 (11 clones) was clustered into Marine Group I with a *g23* sequence (AAZ17585) from the Pacific Ocean (**Figure 6A**). In previous studies, Marine Group I had a wide geographical distribution and was present in samples collected from various marine environments including the north-eastern Gulf of Mexico, north-eastern Pacific and the Arctic Ocean hydrothermal vent, Chesapeake Bay, and Delaware Bay (Filée et al., 2005). Furthermore, OTU PC-148 (six clones) was associated with Marine Group III, companied with *g23* sequences (AAZ17576, AAZ17573, and ABV27498) from seawater, which was mainly observed in Pacific Ocean samples (Filée et al., 2005).

None of the *g23* OTUs from sediments of the PRE belonged to the T+Pseudo group, even though this group was observed in both marine water and paddy soils in previous studies (Filée et al., 2005; Wang et al., 2009b). Hosts of viruses of the T+Pseudo group comprised enterobacteria that inhabited the intestines of mammals and some *Aeromonas* (Tetart et al., 2001) and have not been observed in any studies of benthic microbial communities of the PRE (Liu et al., 2014). The lack of hosts might explain the absence of the T+Pseudo group in this study. However, we found the presence of Exo-T even that consists of viruses infecting cyanobacteria (cyanophages) (Desplats and Krisch, 2003; Filée et al., 2005) in all sites, which indicated that cyanophages might be widespread in the sediments of the PRE. Previous studies have reported that there were cyanophages and phycoviruses in the sediments from the Saanich Peninsula (Canada) (Suttle, 2000; Lawrence et al., 2002). Because relatively few cyanobacteria were observed in PRE sediments (Liu et al., 2014) and they are not supposed to be active in sediment environments, although abundant and active cyanobacteria exist in the column of PRE (Zhou et al., 2004; Owen, 2005; Jiang et al., 2009; Qiu et al., 2010), the viruses were found to belong to the Exo-T-even group and had probably come from sinking particles or infected host cells from the water column.

## Conclusion

The PRE is an important ecosystem linking mainland China and South China Sea, and terrestrial inputs from the Pearl River have a significant influence on estuarine sediments. In this subtropical estuary, TEM analysis showed that the morphology of the viriobenthos was diverse and included tailed, spherical, filamentous, and rod-shaped viruses. Viral and prokaryotic abundances and the diversity of the T4-type phages decreased from freshwater sediments (river mouth) to saltwater sediments (open sea), suggesting that the discharge from the Pearl River had a strong influence on the viral population size in estuarine sediments. In this study, we analyzed the major capsid gene (*g23*) to supplement the sparse information about the sedimentary T4-type phages. Phylogenetic analysis indicated that in estuarine sediments, most of the T4-type phages were from the water column and the relative abundances of marine origin and terrigenous origin viruses differed increasingly between sites. All these results showed that viral communities were mainly influenced by the input of the Pearl River that became less influential with the increasing distance from the river mouth.

## Author Contributions

RZ and NJ conceived and designed the experiments and MH and LC performed the experiments. RZ and CZ contributed the sampling vessel and tools and MH analyzed the data. MH, LC, RZ, CZ, and NJ wrote the paper.

## Conflict of Interest Statement

The authors declare that the research was conducted in the absence of any commercial or financial relationships that could be construed as a potential conflict of interest.
