# Supplementary material for: Phylogenetic Diversity of T4-Type Phages in Sediments from the Subtropical Pearl River Estuary
Source: Front Microbiol. 2017 May 18;8:897. doi: 10.3389/fmicb.2017.00897 (PMC5436276; doi:10.3389/fmicb.2017.00897)
Supplement: Supplementary file 1 [file Table_1.DOC]

Supplementary Material

# Phylogenetic diversity of T4-type phages in sediments from the subtropical Pearl River Estuary

Maoqiu He^a^, Lanlan Cai^a^, Chuanlun Zhang, Nianzhi Jiao^*^, Rui Zhang^*^

*** Correspondence:**Rui Zhang
[ruizhang@xmu.edu.cn](mailto:ruizhang@xmu.edu.cn)

Nianzhi Jiao
[jiao@xmu.edu.cn](mailto:jiao@xmu.edu.cn)

^a^ The authors contributed equally to this paper.

**Supplementary Table 1. Environmental parameters of sampling stations**

| **Station** | **Longitude (°E)** | **Latitude (°N)** | **Depth (m)** | **Temperature (°C)** | **Salinity** | **DO (mg/L)** |
| --- | --- | --- | --- | --- | --- | --- |
| **A** | 113.572 | 22.903 | 0 | 29.1 | 0.14 | 0.65 |
|  |  |  | Sediment | 29.3 | 0.39 | 0.33 |
| **B** | 113.78 | 22.358 | 0 | 27.9 | 20.87 | 4.19 |
|  |  |  | Sediment | 28.8 | 25.10 | 0.27 |
| **C** | 113.936 | 21.213 | 0 | 29.2 | 36.06 | 4.07 |
|  |  |  | 35 | 21.5 | 35.44 | 3.87 |
|  |  |  | 70 | 28.8 | 35.71 | 3.92 |
|  |  |  | Sediment | 25.1 | 31.50 | 0.23 |

**Supplementary Table 2.** Statistical chart of the identities of the closest relatives of *g23* OTUs from sediments of the Pearl River Estuary

| **Station** | **The identity of the closest relatives of *g23* clones** | | | | |
| --- | --- | --- | --- | --- | --- |
|  | **Lowest** | **Highest** | **50~79%** | **80~89%** | **90~100%** |
| **A** | 52% | 100% | 51 (53.1%) | 28 (29.2%) | 17 (17.7%) |
| **B** | 52% | 100% | 56 (69.2%) | 10 (12.3%) | 15 (18.5%) |
| **C** | 50% | 97% | 44 (75.9%) | 5 (8.6%) | 9 (15.5%) |


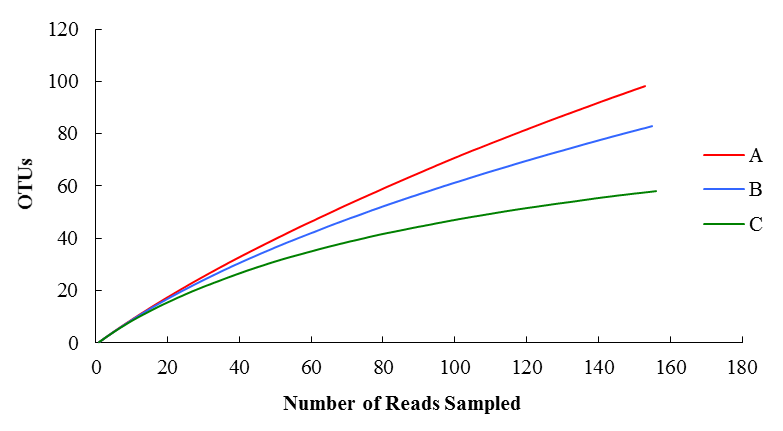


**Supplementary Figure 1.** Rarefaction curve of viral *g23* clone libraries in sediments from the Pearl River Estuary

**Supplementary Figure 2.** Distribution of the 50 most abundant *g23* OTUs in sediments from the Pearl River Estuary

**Supplementary Table 3.** The closest relatives of the 50 most abundant *g23* OTUs in sediments of the Pearl River Estuary at the amino acid level

| **Serial number of OTU** | **Number of clones** | **E value** | **Accession number** | **Identity** | **Sources** |
| --- | --- | --- | --- | --- | --- |
| 1 | 18 | 3.00E-43 | AKZ31873 | 58% | Donghu Lake |
| 2 | 16 | 4.00E-54 | AAZ17601 | 73% | Pacific Ocean |
| 3 | 12 | 1.00E-69 | ABV27498 | 76% | Marine environment |
| 4 | 12 | 2.00E-64 | BAJ61641 | 79% | Black soil in NE China |
| 5 | 11 | 2.00E-42 | AAZ17585 | 59% | Pacific Ocean |
| 6 | 9 | 6.00E-73 | AAZ17576 | 79% | Pacific Ocean |
| 7 | 8 | 3.00E-60 | AAZ17601 | 71% | Pacific Ocean |
| 8 | 8 | 3.00E-48 | BAK52113 | 67% | Paddy soil in NE China |
| 9 | 6 | 1.00E-64 | AAZ17568 | 74% | Pacific Ocean |
| 10 | 6 | 8.00E-55 | AAZ17601 | 66% | Pacific Ocean |
| 11 | 6 | 2.00E-38 | AKZ31902 | 54% | Donghu Lake |
| 12 | 6 | 3.00E-56 | BAF52897 | 70% | Paddy soil in Japan |
| 13 | 6 | 9.00E-76 | AGH68859 | 83% | Kotokel Lake |
| 14 | 6 | 1.00E-44 | AFV99126 | 60% | Chesapeake Bay |
| 15 | 5 | 1.00E-52 | AKZ31900 | 69% | Donghu Lake |
| 16 | 5 | 6.00E-55 | AAZ17601 | 64% | Pacific Ocean |
| 17 | 5 | 5.00E-58 | AAZ17568 | 70% | Pacific Ocean |
| 18 | 5 | 2.00E-54 | AAZ17601 | 66% | Pacific Ocean |
| 19 | 5 | 1.00E-37 | AAZ17601 | 72% | Pacific Ocean |
| 20 | 5 | 7.00E-65 | ABV27498 | 70% | Marine environment |
| 21 | 5 | 3.00E-61 | AMN08858 | 53% | Annecy Lake |
| 22 | 5 | 2.00E-93 | ACZ73359 | 97% | Antarctic Lake |
| 23 | 4 | 1.00E-68 | AFP74264 | 91% | San Pedro Ocean |
| 24 | 4 | 3.00E-82 | AKZ31912 | 98% | Donghu Lake |
| 25 | 4 | 7.00E-62 | BAL45704 | 75% | Wetland soil in NE China |
| 26 | 4 | 7.00E-64 | AAZ17601 | 75% | Pacific Ocean |
| 27 | 4 | 6.00E-41 | AFN85707 | 56% | Dairy wastewater |
| 28 | 4 | 9.00E-58 | AAZ17568 | 68% | Pacific Ocean |
| 29 | 4 | 3.00E-70 | YP_004323491 | 84% | Prochlorococcus phage P-HM2 |
| 30 | 4 | 6.00E-55 | AGH68846 | 61% | Kotokel Lake |
| 31 | 4 | 2.00E-59 | AFV99082 | 76% | Chesapeake Bay |
| 32 | 4 | 2.00E-89 | AFN73188 | 93% | Arctic glaciers |
| 33 | 3 | 7.00E-55 | BAF37238 | 64% | Rice soil in Japan |
| 34 | 3 | 1.00E-81 | AKV56672 | 100% | Sargasso Sea |
| 35 | 3 | 3.00E-72 | AFV99114 | 78% | Chesapeake Bay |
| 36 | 3 | 4.00E-88 | AAZ17576 | 96% | Pacific Ocean |
| 37 | 3 | 2.00E-64 | BAJ61641 | 79% | Black soil in NE China |
| 38 | 3 | 4.00E-59 | BAF52897 | 72% | Paddy soil in Japan |
| 39 | 3 | 8.00E-59 | AFV99082 | 77% | Chesapeake Bay |
| 40 | 3 | 3.00E-83 | AFN73194 | 91% | Kongsfjorden Bay |
| 41 | 3 | 2.00E-35 | AFN85703 | 52% | Dairy wastewater |
| 42 | 3 | 5.00E-68 | ADI87649 | 84% | Donghu Lake |
| 43 | 3 | 6.00E-87 | BAF93261 | 94% | Paddy soil in Japan |
| 44 | 3 | 3.00E-64 | BAJ61641 | 78% | Black soil in NE China |
| 45 | 3 | 9.00E-85 | AKZ31912 | 100% | Donghu Lake |
| 46 | 3 | 3.00E-58 | BAF52897 | 73% | Paddy soil in Japan |
| 47 | 3 | 4.00E-51 | AKZ31900 | 67% | Donghu Lake |
| 48 | 3 | 5.00E-89 | AAZ17576 | 90% | Pacific Ocean |
| 49 | 3 | 1.00E-71 | AKV56661 | 91% | Sargasso Sea |
| 50 | 2 | 6.00E-91 | AKZ31859 | 93% | Donghu Lake |

**Supplementary Table 4.** The closet relatives of the most abundant 10 *g23* OTUs separated in the sampling stations at the amino acid level

| **OTUs** | **Number of clones** | **E value** | **Accession number** | **Identity** | **Sources** |
| --- | --- | --- | --- | --- | --- |
| PA-25 | 12 | 1.00E-62 | BAJ61641 | 78% | Black soil in NE China |
| PA-143 | 8 | 4.00E-47 | BAK52113 | 67% | Paddy soil in NE China |
| PA-160 | 6 | 1.00E-56 | BAF52897 | 71% | Paddy soil in Japan |
| PA-149 | 5 | 2.00E-74 | AGH68859 | 83% | Kotokel Lake |
| PA-151 | 4 | 1.00E-60 | BAL45704 | 75% | Annecy Lake |
| PA-176 | 3 | 4.00E-70 | AHU87119 | 88% | Annecy Lake |
| PA-179 | 3 | 2.00E-64 | BAJ61641 | 79% | Black soil in NE China |
| PA-133 | 3 | 3.00E-63 | BAJ61641 | 79% | Black soil in NE China |
| PA-175 | 3 | 6.00E-68 | ADI87649 | 84% | Donghu Lake |
| PA-123 | 2 | 1.00E-79 | ADI87628 | 89% | Donghu Lake |
| PB-132 | 12 | 8.00E-56 | AAZ17601 | 74% | Pacific Ocean |
| PB-56 | 8 | 1.00E-54 | AAZ17601 | 71% | Pacific Ocean |
| PB-17 | 6 | 5.00E-62 | ABV27498 | 75% | Marine environment |
| PB-194 | 6 | 8.00E-57 | AAZ17568 | 72% | Pacific Ocean |
| PB-15 | 5 | 3.00E-65 | AAZ17576 | 78% | Pacific Ocean |
| PB-167 | 5 | 5.00E-71 | AFV99114 | 78% | Chesapeake Bay |
| PB-165 | 5 | 1.00E-51 | AFV99045 | 64% | Delaware Bay |
| PB-150 | 4 | 4.00E-51 | AAZ17568 | 69% | Pacific Ocean |
| PB-116 | 4 | 2.00E-10 | AFP74466 | 52% | San Pedro Ocean |
| PB-8 | 4 | 1.00E-78 | AAZ17576 | 87% | Pacific Ocean |
| PC-113 | 17 | 4.00E-42 | AFP74283 | 79% | San Pedro Ocean |
| PC-111 | 11 | 2.00E-41 | AAZ17585 | 59% | Pacific Ocean |
| PC-118 | 7 | 8.00E-56 | AAZ17601 | 74% | Pacific Ocean |
| PC-103 | 6 | 3.00E-36 | BAL45721 | 56% | Paddy soil in NE China |
| PC-119 | 6 | 2.00E-43 | AFV99126 | 60% | Chesapeake Bay |
| PC-148 | 6 | 2.00E-59 | ABV27498 | 74% | Marine environment |
| PC-109 | 5 | 1.00E-36 | AHU87197 | 53% | Annecy Lake |
| PC-151 | 5 | 4.00E-92 | ACZ73359 | 97% | Antarctic Lake |
| PC-124 | 4 | 2.00E-65 | AAZ17576 | 78% | Pacific Ocean |
| PC-123 | 4 | 1.00E-49 | AFV99045 | 63% | Chesapeake Bay |
